# Effects of Different Post Warm-Up Strategies for Non-Starter Players in Futsal

**DOI:** 10.5114/jhk/192789

**Published:** 2024-12-19

**Authors:** Nuno Silva, Bruno Travassos, Bruno Gonçalves, Fábio Yuzo Nakamura, Eduardo Abade

**Affiliations:** 1 Research Center in Sports Sciences, Health Sciences and Human Development, CIDESD, University of Maia, ISMAI, Maia, Portugal.; 2 Research Center in Sports Science, Health Sciences and Human Development, CIDESD, University of Beira Interior, UBI, Covilhã, Portugal.; 3 Portugal Football School, Portuguese Football Federation, Oeiras, Portugal.; 4 Sports and Health Department, Health and Human Development, University of Évora, Évora, Portugal.; 5 Comprehensive Health Research Centre (CHRC), University of Évora, Évora, Portugal.; 6 Research Center in Sports Sciences, Health Sciences and Human Development, CIDESD, University of Trás-os- Montes e Alto Douro, UTAD, Vila Real, Portugal.; 7 Departament of Sports Sciences, Exercise and Health, University of Trás-os-Montes and Alto Douro, UTAD, Vila Real, Portugal.

**Keywords:** potentiation, running, jumping

## Abstract

Futsal warm-ups are crucial pre-match routines designed to enhance players’ readiness. However, non-starter players inevitably face longer periods of inactivity. This study aimed to investigate the effects of various post warm-up strategies on physical performance of non-starter players in futsal. Thirteen highly trained male futsal players participated in this study during the in-season period. All players performed three distinct post warm-up strategies over consecutive days: REST, dynamic stretching (DYS), and a combination of plyometrics with change of direction drills (PLY-COD). After performing a standard warm-up, players remained inactive for 10 min, mirroring the traditional time window leading up to the start of the match. Subsequently, post warm-up strategies were implemented. Testing included a countermovement-jump, a reactive strength index, 5- and 10-m sprints, and a 505 COD test. Players were tested 10 min after the conclusion of the warm-up and immediately after the post warm-up strategy. The PLY-COD strategy yielded positive effects across all variables. Running performance improved with small to moderate effect in both sprint (−2.2 ± 1.9%), (−1.6 ± 1.7%), and COD (−2.9 ± 3.5%) tests. Conversely, both DYS and REST strategies had a detrimental impact on running and jumping performances, with this impairment being more pronounced in running following REST strategy, particularly in the 10-m linear sprint (1.4 ± 1.7%). These results suggest that remaining inactive or exerting limited effort after a warm-up may be detrimental to physical performance of futsal non-starter players. On the other hand, PLY-COD drills could be effective strategies to maintain or even enhance physical performance following the warm-up.

## Introduction

Futsal is a team sport played in a 5-a-side format on a 40 m x 20 m court, in which coaches can make unlimited substitutions during an official match. Regular substitutions are a key factor in enabling players to maintain high-intensity actions throughout the match and are particularly important in ensuring players are at their best when entering the match (Ribeiro et al., 2023). Given that only five players start the match and non-starter players remain inactive until their participation, and considering that a general decrease in players’ readiness to perform could occur during this time (Crowther et al., 2017; Galazoulas et al., 2012), there is a need to implement both passive and active post-warm-up strategies. Therefore, a deeper understanding of the impact of passive and active post-warm-up strategies in futsal is required.

Passive strategies aim to maintain muscle temperature during the post-warm-up period, delaying the negative effects of passive rest (McGowan et al., 2015), while active strategies could encompass a range of activities such as stretching, running, jumping, cycling exercises or performing high-intensity actions like resistance exercises (McGowan et al., 2015). Some research also suggests the use of combined passive and active post-warm-up strategies to maximize players’ readiness (West et al., 2016) and several strategies can be considered by coaches, although stretching exercises combined with changes of direction or plyometric exercises had been considered to be most suitable as post-warm-up strategies (Abade et al., 2017) due to their simplicity and potential to increase players’ readiness. However, consensus is still lacking regarding the best post-warm-up strategies to maintain players’ readiness to perform in futsal.

While the importance of post warm-up (WU) strategies is widely recognized and supported by research (Silva et al., 2018), questions remain about the most suitable activities and structure of the post WU. When designing a post WU, professionals should consider the official rules of competition and the specific dynamics of the sport. For instance, in futsal, it is crucial to understand that there is limited space for players to perform a post WU, and only five players are permitted to do so at the same time. These constraints align with the interchange dynamics of rotation in an elite futsal match, taking into consideration each coach’s strategy (Ribeiro et al., 2024). However, it is evident that a regular pace of substitutions helps maintain a high intensity in players’ performance throughout the match (Dos-Santos et al., 2020; Ribeiro et al., 2022). Therefore, there is a need to test and explore the best strategies that enable players to enhance their readiness to perform when entering the match. This study aimed to investigate the effects of different post WU strategies (rest, dynamic movements, and a combination of plyometric and change of direction (COD) movements) on the readiness of non-starter players in futsal. It was hypothesized that a post WU strategy combining plyometric and COD exercises could improve the readiness of non- starter players in futsal.

## Methods

### Participants

Thirteen highly trained (McKay et al., 2022) male futsal players (22.5 ± 5.6 years, 1.82 ± 0.08 m, 76.1 ± 5.4 kg) from a Portuguese first division futsal team participated in this study. The study was conducted during the in-season period, with a weekly schedule of five on-court training sessions (approximately 90 min each in the evening) and an official match on the weekend. Goalkeepers were excluded from the study due to the unique rotational dynamics of goalkeeping in a futsal match. All players were informed about the study design, requirements, and procedures, and their consent was obtained. Players were invited to participate in the study 15 days before its commencement and were free to withdraw at any time. The investigation was approved by the Ethics and Scientific Committee of the University of Beira Interior (approval code: CE-UBI-Pj-2020-043; approval date: 19 May 2020) and adhered to the 1964 Helsinki declaration.

### Measures

**Figure 1:**
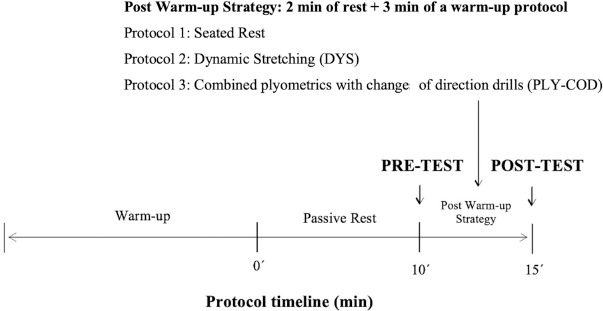
Schematic representation of the experimental design. Both pre-test (10 min after the end of the warm-up) and post-test (immediately after the post warm-up strategy) evaluation tests were included to assess differences in protocols.

Assessments were conducted at the same time of day (8 pm) in an indoor facility with an official match surface and similar environmental conditions (22º–23º Celsius). Pre-test measures were recorded 10 min after the end of the standard warm-up (WU) (Figure 1). Post-testing occurred immediately after the end of the post WU protocol. Given that futsal requires players to perform repeated short-term high-intensity actions such as jumping, sprinting, and changes of direction (Spyrou et al., 2020), testing included a countermovement jump (CMJ), a reactive strength index (RSI), linear sprints (of 5 m and 10 m) and a change of direction speed test (505).

The CMJ test and a depth jump for the RSI calculation were performed using an Optojump device (Microgate, Italy). The CMJ was performed according to previous guidelines (Abade et al., 2017). Participants were asked to stand upright with their hands on their hips. When ready, the athlete squatted down until their knees were bent at 90 degrees, then immediately jumped vertically as high as possible, while keeping their hands on their hips. To evaluate the RSI, players were asked to perform a depth jump from a 20-cm box and instructed to jump as high as possible with minimal ground contact time. The RSI was calculated using the following formula: RSI = Jump Height / Ground Contact Time. This test evaluates the ability of players to rapidly transition from an eccentric motion to a concentric contraction, an ability that appears to be associated with maximal horizontal deceleration ability (Jarvis et al., 2022). Participants performed two maximal attempts for each jump with a 30-s rest interval in between. The best trial was recorded for subsequent analysis.

Linear sprint (5-m and 10-m) and 505 tests were evaluated in a single all-out trial. For this purpose, three infrared timing gates (Witty, Microgate, Italy) were placed at a starting line, and at the 5^th^ and the 10^th^ m. For the 505 test, participants began 0.5 m behind the starting line and were asked to run 15 m before performing a 180° change of direction (marked by a turning point) with the dominant leg. The time was recorded from when the player first crossed the 5-m marker and stopped when the player crossed the same marker again (5 m up and back, for a total of 10 m). During the 505 test, linear 5-m and 10-m split times were recorded. Two trials were allowed with a 90-s rest interval between trials.

Sprint and 505 tests were performed 30 s after the jumping tests and players were evaluated in sequential order to ensure equal time of recovery for all participants.

### Design and Procedures

A repeated measures design was employed to evaluate three post warm-up (WU) strategies in a randomized sequence over three consecutive days. All players participated in the same protocol each day. For all protocols, the intervention began with a specific futsal WU based on an elite WU model (Silva et al., 2020). After the WU, a 10-min passive rest was provided to mimic a typical competitive pre-match scenario. Pre-testing occurred immediately after the passive rest. Subsequently, a 5-min window was used to represent the rotational dynamics of an elite futsal team (Ribeiro et al., 2022), consisting of a 2-min rest and 3-min intervention. During the REST protocol, players remained seated for 5 min. In the DYS protocol, players were instructed to perform various dynamic stretches of the lower limbs. Finally, the PLY-COD strategy combined plyometric exercises with a change of direction (COD) drill. Post-testing was conducted immediately after the end of the post WU protocol. This timeline simulated a real-world scenario, representing the passive rest time between the end of the WU and the additional time a non-starter player may wait before entering the match.

The three intervention protocols were applied in a small area behind the bench in accordance with futsal rules. The REST protocol consisted of players remaining seated during the 5-min intervention. In the DYS intervention, players were instructed to perform three sets of five repetitions of the following exercises: bilateral squats, hip extension, hip flexion, hip adduction, and hip abduction, sequentially with 30-s rest intervals between sets. Each unilateral exercise was performed with both legs. The PLY-COD protocol consisted of three sets of the following drills: 2-m distance low skipping, three bilateral jumps, and acceleration with two COD turns of 45º (one for each side) at maximum intensity. Players were instructed to rest for 30 s between subsequent sets.

### Statistical Analyses

An estimation technique approach was applied to overcome the shortcomings associated with traditional N-P null hypothesis significance testing (Cumming, 2012; Ho et al., 2019). Estimation plots for running performance, vertical jump and 505 tests were used as descriptive statistics according to the REST, DYS and PLY-COD protocols. This graphical representation shows the individual values for pre- and post-test measures and the mean difference with 95% of confidence intervals (CIs) (Cumming, 2012; Ho et al., 2018). The day-to-day variability (within-players) in pre-test performance was measured as typical error and expressed as a coefficient of variation, CV (%) (Hopkins, 2000). The % of variation from pre- to post-test was computed for all variables and used as a dependent variable to determine which protocol(s) might favour performance. Cohen’s *d*_unbiased_ (*d*_unb_) with 95%CI (an unbiased estimate has a sampling distribution of which the mean equals the population variable being estimated) was applied to identify pairwise differences among all previous comparisons (Cumming, 2012). The pooled with-in groups SD was used to calculate *d*. Thresholds for effect size statistics were: 0.2, 0.5, and 0.8 for small, medium, and large effect size, respectively (Cohen, 1988).

## Results

**Figure 2:**
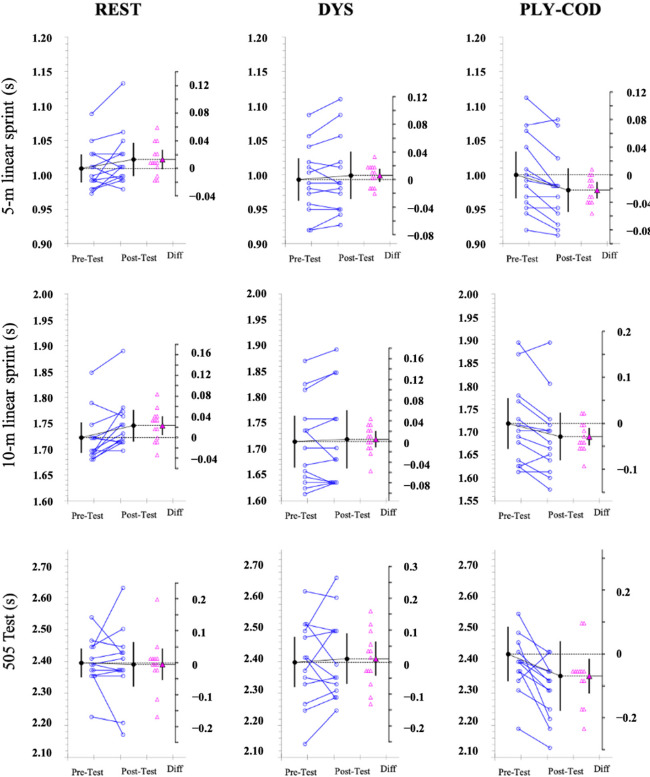
Paired data showing means and 95% confidence intervals of pre-test and post-test measures for the 5-m linear sprint, the 10-m linear sprint and the 505 test according to each protocol. The mean paired difference is shown with its 95% confidence interval against a floating difference axis, of which zero is lined up with the pre-test mean. The paired data are shown as small circles joined by lines. The differences are shown as triangles on the difference axis.

**Figure 3:**
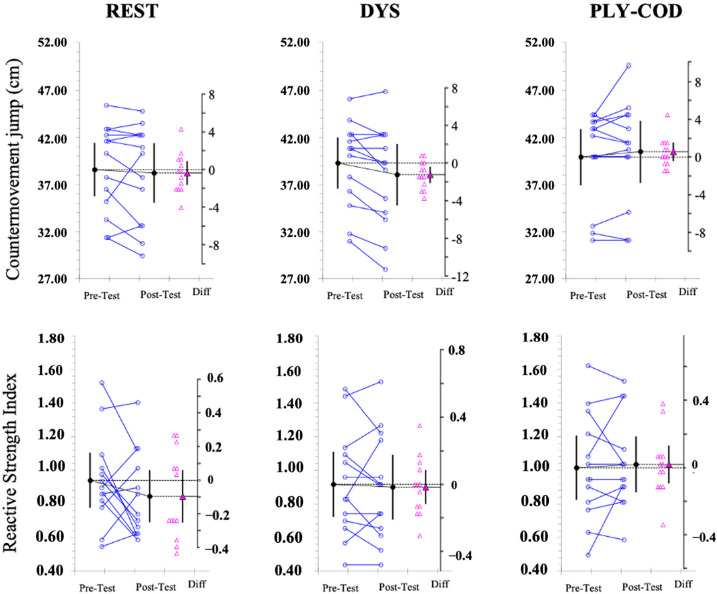
Paired data showing means and 95% confidence intervals of pre-test and post-test measures for the countermovement jump and the reactive strength index according to each protocol. The mean paired difference is shown with its 95% confidence interval against a floating difference axis, of which zero is lined up with the pre-test mean. The paired data are shown as small circles joined by lines. The differences are shown as triangles on the difference axis.

The summary of the descriptive and inferential analysis is presented in Table 1. Individual and differences of mean values from pre- to post-test performance measures are shown in Figures 2 and 3. The estimation plots present the differences of means as a bootstrap 95% confidence interval on separate but aligned axes. This representation can be visualized for each variable and protocol.

**Table 1:** Descriptive results (mean ± SD) for each variable according to warm-up protocols and inferential results for within comparison of the percentage variation from the pre- to post-test moments.

Variables	REST		
	Pre	Post	Cohen’s *d_unb_* [95% CI]
5-m linear sprint, s	1.01 ± 0.03	1.02 ± 0.04	0.33 [−0.03, 0.72]
10-m linear sprint, s	1.72 ± 0.05	1.75 ± 0.05	0.43 [0.08, 0.83]
505 test (505), s	2.40 ± 0.07	2.39 ± 0.12	−0.04 [−0.51, 0.42]
Countermovement jump (CMJ), cm	38.58 ± 4.70	38.23 ± 5.22	−0.07 [−0.30, 0.16]
RSI Index (RSI)	0.94 ± 0.27	0.84 ± 0.26	−0.34 [−0.90, 0.20]
	DYS		
5-m linear sprint, s	0.99 ± 0.05	1.00 ± 0.06	0.11 [−0.05, 0.27]
10-m linear sprint, s	1.71 ± 0.08	1.72 ± 0.09	0.05 [−0.11, 0.21]
505 test (505), s	2.40 ± 0.13	2.41 ± 0.13	0.08 [−0.29, 0.45]
Countermovement jump (CMJ), cm	39.27 ± 4.51	38.04 ± 5.42	−0.23 [−0.43, -0.06]
RSI Index (RSI)	0.92 ± 0.32	0.90 ± 0.32	−0.05 [−0.34, 0.23]
	PLY-COD		
5-m linear sprint, s	1.00 ± 0.06	0.98 ± 0.05	−0.38 [−0.66, −0.15]
10-m linear sprint, s	1.72 ± 0.09	1.69 ± 0.09	−0.30 [−0.55, −0.08]
505 test (505), s	2.42 ± 0.14	2.35 ± 0.18	−0.40 [−0.77, −0.07]
Countermovement jump (CMJ), cm	39.91 ± 4.95	40.48 ± 5.47	0.10 [−0.07, 0.28]
RSI Index (RSI)	1.02 ± 0.32	1.04 ± 0.28	0.06 [−0.28, 0.41]

REST: Seated rest protocol; DYS: Dynamic stretching protocol; PLY-COD: Plyometric and change of direction protocol; d_unb_: d_unbiased_; CI: confidence interval

The inter-day (within-players) pre-test players’ performance presented ~10% variation in jumping tests (3.7 ± 2.6 in the CMJ and 16.4 ± 9.1 in the RSI) and ~3% variation in running tests (3.3 ± 2.1 in the 5-m, 2.8 ± 1.7 in the 10-m and 3.0 ± 2.1 in the 505 test).

For the REST protocol, a general decrement in players’ performance in all variables was identified, with the exception of the 505 test, from pre- to post-test. There was a 1.3 ± 2.3% increase in the 5-m linear sprint test, a 1.4 ± 1.7% increase in the 10-m linear sprint test (pre- to post-test mean difference with 95% CI = 0.43 [0.08, 0.83]; with moderate ES), and an unclear pattern in jumping testing from pre- to post-test in the 505 test with a decrease of 0.20 ± 3.26%, a 0.95 ± 5.7% decrease in the CMJ and a 6.35 ± 27.8% increase in the RSI.

For the DYS protocol, there were trivial decrements in players’ performance from pre- to post-test in all variables, except for the CMJ. A 0.6±1.5% increase was observed in 5-m linear sprint, a 0.3 ± 1.5% increase in 10-m linear sprint and a 0.5 ± 3.5% increase in 505 test times. As aforementioned, the CMJ decreased by 3.4 ± 3.7% from pre- to post-test (−0.23 [−0.43, −0.06], small ES), while the RSI displayed a decrease of −0.5 ± 18.5%.

The PLY-COD strategy showed positive effects in the 5-m linear sprint (−2.2 ± 1.9% (−0.38 [−0.66, −0.15]; with small to moderate ES), the 10-m linear sprint (−1.6 ± 1.7% (−0.30 [−0.55, −0.08]; with small ES) and the 505 test (−2.9 ± 3.5% (−0.40 [−0.77, −0.07]; small to moderate ES). Regarding jumping performance, unclear changes for both the CMJ and the RSI were noted.

**Figure 4:**
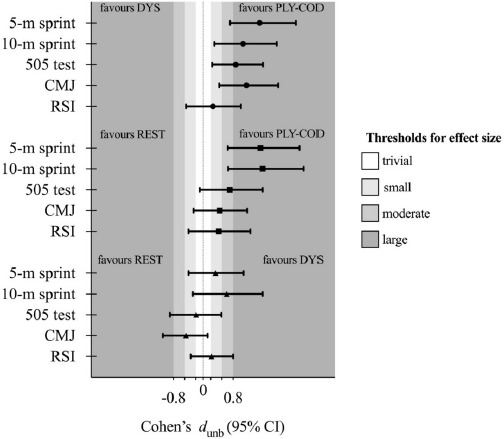
Cohen’s *d*_unbiased_ for within comparison of the % variation from the pre- to post-test protocols. Error bars indicate uncertainty in true mean changes with 95% confidence intervals. Note: Since higher negative % of variation from the pre- to post-test in the 5-m sprint, the 10-m sprint and the 505-time test is related with better performance, the corresponding outcomes were changed from negative to positive, and vice-versa.

Finally, Table 2 presents the descriptive and inferential results for the % of change in the mean from pre- to post-test comparisons. Figure 4 presents the Cohen’s *d*_unbiased_ (*d*_unb_) with 95% confidence intervals and can be used to depict which protocol favored performance in jumping and running variables. Overall, and contrasting the effects of each protocol, REST and DYS strategies presented unclear differences between each other for jumping and running tests, with trivial effects. Comparing REST and PLY-COD protocols, linear sprinting was favored by PLY-COD strategy with large ES and a positive trend in the other variables. In addition, when comparing DYS and PLY-COD protocols, the latter presented better results with large ES in all variables with the exception of the RSI.

**Table 2:** Descriptive and inferential results for within comparison of the % variation from the pre- to post-test protocols.

Variables	REST	DYS	PLY-COD	Cohen’s *d_unb_* [95% CI]		
				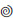 REST vs. 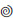 DYS	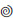 REST vs. 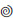 PLY-COD	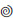 DYS vs. 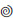 PLY-COD
5-m linear sprint, s	1.30 ± 2.25	0.60 ± 1.48	−2.20 ± 1.85	−0.33 [−1.08, 0.39]	−1.53 [−2.58, −0.66]	−1.51 [−2.48, −0.72]
10-m linear sprint, s	1.35 ± 1.68	0.25 ± 1.46	−1.63 ± 1.69	−0.63 [−1.59, 0.28]	−1.59 [−2.69, −0.67]	−1.07 [−1.97, −0.30]
505 test (505), s	−0.20 ± 3.26	0.51 ± 3.50	−2.89 ± 3.51	0.19 [-0.49, 0.89]	−0.71 [−1.59, 0.09]	−0.87 [−1.6, −0.24]
Countermovement jump (CMJ), cm	−0.95 ± 5.67	−3.40 ± 3.70	1.37 ± 3.71	−0.46 [−1.08, 0.12]	0.44 [−0.26, 1.18]	1.16 [0.43, 2.01]
RSI Index (RSI)	−6.35 ± 27.84	−0.47 ± 18.47	5.73 ± 24.06	0.22 [−0.33, 0.80]	0.42 [−0.39, 1.26]	0.26 [−0.46, 1.01]

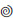
REST: % variation from the pre- to post-test in the seated rest protocol; 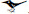
DYS: % variation from the pre- to post-test in the dynamic stretching protocol; 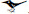
PLY-COD: % variation from the pre- to post-test in the plyometric and change of direction protocol; d_unb_: d_unbiased_; CI: confidence interval

## Discussion

This study aimed to investigate the effects of different post warm-up (WU) strategies on the readiness of non-starter players in futsal. As anticipated, the use of varied post WU strategies may have different effects on the physical readiness of non-starter futsal players when entering the match. Remaining seated or performing activities with limited effort, such as dynamic stretching movements (DYS), after the WU could have trivial or even detrimental effects on physical performance of non-starter players. In contrast, the use of short-term, more intense activities such as plyometric and change of direction (PLY-COD) drills may maintain or even improve physical performance during the transition period between the end of the WU and the moment the player enters the match. Importantly, players responded differently to the same post WU strategy, highlighting the need to select appropriate strategies according to each player’s individual profile.

When analyzing futsal post WU practices in a competitive scenario, professionals often choose to have non-starter players engaged in passive activities, such as seated rest, while waiting to enter the match. The reasoning behind this choice may be related to the lack of applied studies supporting different strategies, as well as the influence of certain empirical practices in team sports competitive contexts. However, the results of this study, in line with previous research in other sports, clearly showed that keeping non-starter players seated might be a questionable option, as it was found that physical performance variables decreased at the post-test moment, particularly the linear running capacity. These data are consistent with previous research that shows a trend of performance impairment in the minutes following the end of the WU. This decrease becomes quite evident shortly after the first 6–10 min, and continues its downward trend over time (Crowther et al., 2017; Galazoulas et al., 2012). Results can be explained by the decline in muscular temperature, which is essential for the execution of high-intensity actions that characterize futsal, as well as the return of the heart rate to baseline levels (Alberti et al., 2014; Galazoulas et al., 2012; McGowan et al., 2015). This means that this decline in performance may occur not only with non-starter players, but also with starter players if coaches unbalance the work-rest ratio time of each player over the match duration (Ribeiro et al., 2022).

Different post warm-up (WU) strategies can indeed have varying effects (Crowther et al., 2017), and it is common to observe non-starter players standing up and/or performing some dynamic stretching movements while waiting to enter the match. However, the execution of these low-intensity dynamic activities seems to be insufficient to prevent performance impairment during the transition period between the end of the WU and the moment they actually start to perform in the game. The results of the present study showed a trivial effect on most variables, with a tendency towards a decrease in performance which was more evident in the jumping variables. Thus, it appears that dynamic stretching exercises are likely insufficient to elevate players’ muscular temperature to levels similar to those at the end of the WU. However, this hypothesis needs to be tested in the future.

Previous research has revealed that dynamic stretching shows superior acute effects compared to static stretching (Zmijewski et al., 2020), particularly on the range of motion and the connection between the tendon and muscle which is associated with stronger contractions promoted by high intensity activities (Opplert & Babault, 2019), to improve players readiness to perform. However, combination of dynamic stretching with other exercises remains unclear due to divergent results obtained (Blazevich et al., 2018; Moreno-Perez et al., 2021; Yamaguchi et al., 2019), and the possible potentiating effect for subsequent activities continues to be debatable. This acute potentiating effect is typically associated with more intense activities, attributed to the increased potential for cross-bridge formation in the muscle and the corresponding enzymatic activity (Opplert and Babault, 2018).

After carefully examining the results and strategies used, it seems possible to conclude that a post WU involving short-term but intense actions may be more beneficial for non-starter players in a futsal match. In fact, the post WU strategy that included plyometric and change of directions exercises demonstrated potential for enhancing players’ performance, particularly in running variables, both linear and with changes of direction. A positive trend was also observed in jump variables, although with a lower effect size.

The literature shows that longitudinal plyometric training programs may lead to benefits in different physical capacities, such as jumping, running and sprinting (Kons et al., 2023; Slimani et al., 2016). In addition, positive acute effects of combining plyometric and COD strategies have been noted (Abade et al., 2017). In this regard, plyometrics might improve the post WU due to the involvement of the muscle’s stretch-shortening cycle and its potential to improve the nerve reflexes associated with higher motor unit recruitment (Andrade et al., 2015). In addition to the potentiation in the concentric phase, it is essential to consider the eccentric force absorption phase, in which there is a peak force associated with landing, which is extremely demanding to the neuromuscular system (Ebben et al., 2011). Thus, it is accepted that plyometric exercises may increase body and muscle temperature, concurrently with enhancement of motor unit firing frequency and synchronization, thus promoting short-term performance improvement (Slimani et al., 2016). As mentioned before, plyometric exercises involve high eccentric impact and concentric propulsion. Meanwhile, change-of-direction activities include a deceleration, rapid stabilization, and a propulsion phase. Therefore, there seems to be an evident potentiation effect of performing plyometrics on the force absorption and propulsive abilities that will be decisive in the braking, stabilization and acceleration phases of change-of-direction movements (Dos'Santos et al., 2017).

Even with the limited time and space available for post WU interventions, it is crucial that the motor pattern of the conditioning activities closely resembles the most relevant actions in the game. Notably, movements in the plyometric and change of direction (PLY-COD) strategy are representative of actions directly linked to decisive movements in the game, such as shooting, changing direction, accelerating, and decelerating. These movements, in addition to their physical dimension, may also establish an adequate “mindset” for competition. Previous research highlights more evident optimization when there is a connection between conditioning activities and motor patterns used in the competitive play, as opposed to, for example, cycling movements (Cormier et al., 2022).

Furthermore, significant individual variability in all strategies was observed (Figure 2). Therefore, caution should be applied when interpreting these results, and coaches should consider this aspect to outline the best strategy for each player and thus optimize the post WU activities for non-starter players. Although the limited number of participants in our study may be perceived as a limitation, it is important to note that this is reflective of the inherent constraints encountered when collecting data in elite athletes’ populations, thus providing a realistic representation of the challenges faced in such contexts. Further research is required to evaluate the impact of such strategies as the match progresses in duration and under repetitive rotations.

## Conclusions

The use of different post WU strategies promoted different effects in futsal players and should be considered at the moment when non-starter players approach to enter the match. Taking into consideration the results of this study, remaining seated may not be the most adequate strategy to apply, as well as dynamic stretching may need some high-intensity exercises to complement in order to achieve a potentiation effect. However, coaches may apply active post WU strategies based on plyometric combined with change of direction exercises. These activities can be organized in small spaces and with limited material, which makes them ideal to use in a futsal competitive match context.
